# Neurophysiological Predictors of Proximal Motor Rehabilitation in Stroke Patients with Corticospinal Tract Damage

**DOI:** 10.3390/brainsci16050505

**Published:** 2026-05-08

**Authors:** Wen Dai, Qun Zhang, Jing Tian, Shouyan Wang, Rongrong Lu

**Affiliations:** 1 Institute of Science and Technology for Brain-Inspired Intelligence, State Key Laboratory of Medical Neurobiology and MOE Frontiers Center for Brain Science, State Key Laboratory of Brain Function and Disorders, Fudan University, Shanghai 200433, China; daiwen@fudan.edu.cn (W.D.);; 2 Key Laboratory of Computational Neuroscience and Brain-Inspired Intelligence (Fudan University), Ministry of Education, Shanghai 201213, China; 3 Zhangjiang Fudan International Innovation Center, Shanghai 201213, China; 4 Department of Rehabilitation Medicine, Huashan Hospital, Fudan University, Shanghai 200040, China; zqun_888@163.com (Q.Z.);

**Keywords:** stroke, transcranial magnetic stimulation, motor-evoked potential, intracortical inhibition, motor rehabilitation

## Abstract

**Highlights:**

**What are the main findings?**

• Contralesional neurophysiological measures alone showed limited predictive value in severe corticospinal tract injury
• A combination of contralesional indicators showed a potential association with proximal upper-limb motor improvement after rehabilitation.

**What are the implications of the main findings?**

• The findings of this study are consistent with, but do not directly demonstrate, compensatory contributions from the contralesional cortico-reticulo-spinal pathway in motor recovery.

**Abstract:**

**Background/Objectives:** Upper-limb motor dysfunction is common after stroke, and patients often have limited recovery during rehabilitation. In this study, we aimed to investigate the relationship between contralesional neurophysiological parameters and the effects of rehabilitation on upper-limb motor function in stroke patients with corticospinal tract damage. **Methods:** Forty patients with subacute stroke with an absent MEP response on the ipsilesional side before admission were included. Contralesional neurophysiological parameters, including resting motor threshold, contralesional MEP, contralesional short-interval intracortical inhibition (short-ICI), and contralesional long-interval intracortical inhibition (long-ICI), were assessed via transcranial magnetic stimulation (TMS) pre-admission. The coefficients of variation for MEP, short-ICI, and long-ICI were calculated to assess cortical stability. Rehabilitation effect was measured using changes in the Fugl–Meyer assessment score after 21 days of rehabilitation. **Results:** No single contralesional parameter significantly predicted rehabilitation effect. Further exploratory analysis revealed that a model combining contralesional neurophysiological parameters was associated with proximal limb motor function recovery. Short-ICI played a prominent role in this exploratory model. **Conclusions:** Contralesional neurophysiological markers demonstrated limited predictive value in patients with stroke with moderate-to-severe motor dysfunction and damaged corticospinal tract function on the ipsilesional side. However, a model combining multimodal contralesional TMS measures, particularly short-ICI, may offer incremental value in predicting proximal limb motor improvement following 21-day rehabilitation. Although this mechanism was not directly measured, the findings suggest a compensatory role of the cortico-reticulo-spinal pathway. These exploratory results should be interpreted with caution regarding their clinical applicability and are premature as a predictive tool, pending rigorous external validation.

## 1. Introduction

Stroke is the leading cause of disability worldwide [1]. Stroke patients often experience diverse movement disorders, some of which resolve within days to weeks, whereas others persist and lead to permanent disability that markedly impairs activities of daily living [2]. Upper-extremity motor dysfunction is among the most common sequelae of stroke [3]. Many stroke patients with upper-limb dyskinesia participate in rehabilitation programs yet achieve limited functional recovery. Fewer than half of stroke patients regain meaningful functional use after upper-limb hemiparesis [4]. Therefore, predicting functional recovery before rehabilitation can guide clinicians toward appropriate treatment strategies and reduce both time and economic burdens.

Motor-evoked potentials (MEPs) elicited by transcranial magnetic stimulation (TMS) are reliable clinical markers for assessing and predicting upper-limb motor recovery after stroke [5,6]. MEP amplitude reflects the strength of corticospinal synaptic transmission from primary motor cortex (M1) neurons to spinal motor neurons and the capacity to activate target muscles [7]. Accordingly, the MEP provides an index of corticospinal tract integrity and excitability [8]. The absence of MEP is considered to indicate damage to the corticospinal tract.

Ipsilesional (affected side) MEP amplitude correlates with motor impairment severity as measured on a clinical scale such as the Fugl–Meyer Assessment (F-M) and the Action Research Arm Test [9,10]. Patients with detectable ipsilesional MEP have a better prognosis than those without ipsilesional MEP [10,11,12]. Although absent ipsilesional MEP in the acute and subacute phases can indicate severe motor dysfunction and a poorer prognosis, this relationship is not absolute. Some patients without ipsilesional MEP still achieve meaningful motor recovery with rehabilitation [13,14,15]. Therefore, predicting the prognosis of patients without ipsilesional MEP remains an important topic.

In patients without ipsilesional MEP, changes in the contralesional (unaffected side) MEP amplitude can also be used as biomarkers to predict stroke recovery [16]. However, contralesional MEP alone is not effective in predicting prognosis. The specificity and sensitivity of prognosis prediction using contralesional MEP amplitude alone are only 70–80% [11]. Therefore, integrating contralesional MEP with other neurophysiological parameters on the contralesional side can more accurately predict the recovery of motor function in stroke patients without ipsilesional MEP.

Intracortical inhibition (ICI) is another important TMS marker utilized for evaluation of motor function recovery after stroke [17]. This represents the major inhibitory circuit in the M1. Short-ICI occurs when subthreshold conditioning stimulation suppresses the MEP induced by suprathreshold test stimulation at interstimulus intervals of 1–5 ms [18]. Long-ICI is observed when a superthreshold conditioning stimulation precedes the test stimulation at intervals of 50–200 ms. Short- and long-ICI are mediated by γ-aminobutyric acid type A and type B receptor activity, respectively [8,19]. As a major inhibitory neurotransmitter, γ-aminobutyric acid plays a key role in the modulation of motor cortex plasticity [20]. Furthermore, γ-aminobutyric acid (GABA)-mediated tonic neuronal inhibition in the peri-infarct zone of mice after stroke has been reported [21]. Abnormalities in GABA-mediated intracortical inhibition, involving both short- and long-ICI, have also been reported in humans [22,23,24]. In the acute and subacute phases of stroke, patients show significantly higher intracortical inhibition (i.e., reduced inhibition) on the ipsilesional side than on the contralesional side, and higher bilateral intracortical inhibition than healthy age-matched controls [25,26,27]. These results suggest reduced intracortical inhibition of M1 after stroke. Moreover, as rehabilitation progresses, changes in bilateral ICI show a positive linear correlation [28]. Changes in ICI are also positively correlated with improvements in multiple clinical scores of motor function, such as the F-M and Ashworth scale [29]. ICI is also an important index for evaluating the excitation–inhibition balance in the M1 in several stroke studies [30,31]. These results indicate the potential complementary role of ICI as a parameter with MEP in predicting recovery outcomes in stroke patients without ipsilesional MEP.

Therefore, we aimed to measure contralesional MEP and contralesional short- and long-ICI in stroke patients in the subacute phase before motor rehabilitation, and to exclude ipsilesional MEP, to develop a model to predict F-M improvement after 21 days of motor rehabilitation. In the model, we evaluated corticospinal tract excitability and M1 intracortical inhibition by measuring the amplitudes of contralesional MEP and ICI. In addition to the MEP amplitude and ICI ratio, we calculated the coefficient of variation of the three indicators to evaluate the stability of the neurophysiological parameters of the patients [32]. We hypothesized that a model combining contralesional MEP and ICI would be more effective than contralesional MEP alone for predicting early-phase rehabilitation responsiveness in patients with stroke. In addition, combining cortical stability improved the prognostic accuracy compared to focusing only on the degree of corticospinal tract excitability and M1 intracortical inhibition.

## 2. Materials and Methods

### 2.1. Participants

**Figure 1:**
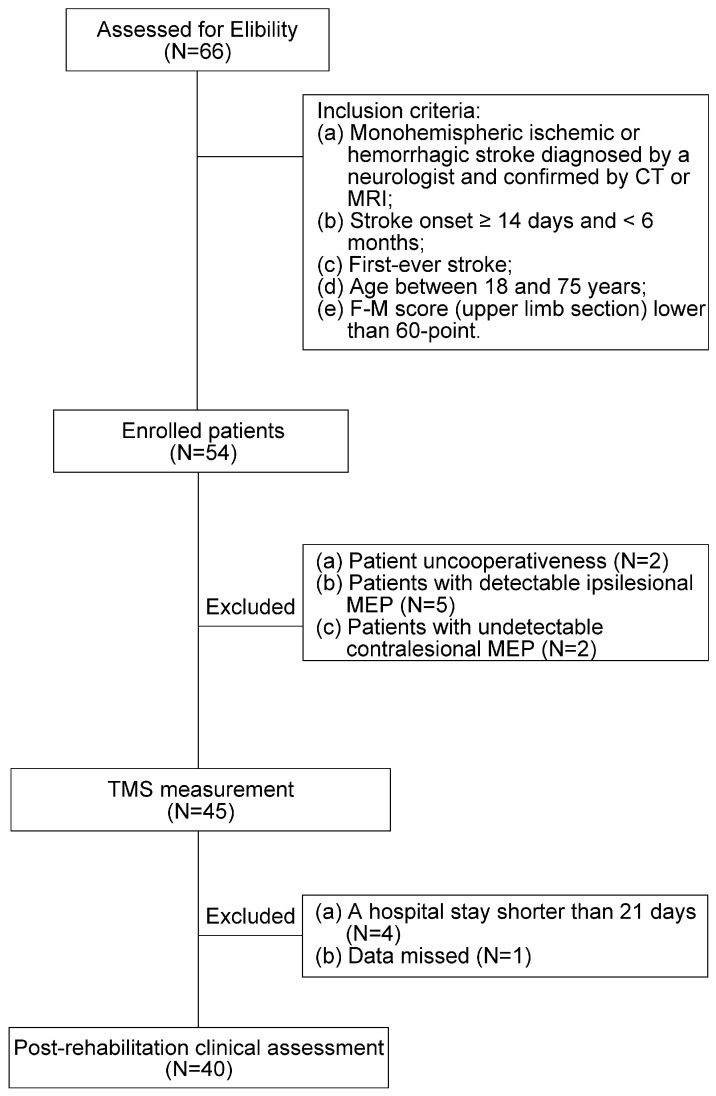
Flow chart of patient enrollment. CT, computed tomography; MRI, magnetic resonance imaging; F-M, Fugl–Meyer Assessment; MEP, motor-evoked potential; TMS, transcranial magnetic stimulation.

Forty patients with stroke (five females, 21 with right-sided hemiplegia, mean age ± standard deviation: 56.60 ± 9.38 years, post-stroke duration: 2.42 ± 2.07 months) were enrolled in this study. As shown in Figure 1, the inclusion criteria were as follows: (i) monohemispheric ischemic or hemorrhagic stroke diagnosed by a neurologist and confirmed by CT or MRI; (ii) stroke onset ≥ 14 days and <6 months; (iii) first-ever stroke; (iv) age between 18 and 75 years; (v) an F-M score lower than 60 points for the affected upper limb [33]; and (vi) absence of ipsilesional MEP. In this study, corticospinal tract damage was defined by the absence of an ipsilesional MEP response. This was an operational, neurophysiological criterion, rather than a measurement from lesion volume or diffusion-based CST methods. We excluded patients with concomitant neuropathies, systemic vasculopathies, epilepsy, or dementia that may render patients uncooperative; presence of intracerebral clips or pacemakers; pregnancy; and hospital stay shorter than 21 days. Supplementary Table S1 shows detailed information for each participant. All participants provided oral informed consent, in accordance with the Declaration of Helsinki. This study was approved by the Ethics Committee of Huashan Hospital (approval number: ChiCTR2300077453).

### 2.2. Rehabilitation Effect

The rehabilitation effect on motor function was evaluated using the upper-extremity section of the F-M, which consists of 10 sub-items: reflex activity, flexor synergies, extensor synergies, movement associated with synergies, movement out of synergy, spasticity, wrist stability, wrist movement, hand function, and coordination/speed. As the cortical areas evaluated by TMS were limited, and we aimed to obtain a clinically interpretable description of recovery patterns, the 10 sub-items of the F-M were further grouped into proximal, distal, and whole limb functions according to the predominant anatomical distribution and motor control demands of the items, which is broadly consistent with the somatotopic organization of M1 and the distinction between proximal arm control and distal dexterity [34,35]. Sub-items 2, 3, 4, and 5 were classified as proximal function, sub-items 7, 8, and 9 as distal function, and sub-items 1, 6, and 10 as whole limb function. This grouping was an anatomy-based approach intended to describe recovery patterns and does not serve as a validated alternative to the standard total F-M score.

All patients were assessed using the F-M at admission (baseline) and after 21 days of comprehensive rehabilitation. Changes in rehabilitation effect were assessed as follows: rehabilitation = F-M_post-rehabilitation_ − F-M_baseline_. According to the minimal clinically important difference for stroke [36], patients with a total rehabilitation change (rehabilitation_total_) of ≥6 were defined as “improvement,” while those with a score < 6 were defined as “non-improvement.” An improvement in a sub-item was defined as a change in the F-M score exceeding 10% of the total score of the sub-item [36]. Therefore, patients with a change in the proximal F-M score (rehabilitation_proximal_) > 4, the distal F-M score (rehabilitation_distal_) >3, and a whole limb F-M score (rehabilitation_whole_) > 1 were defined as improved motor function.

### 2.3. Neurophysiological Parameter

Neurophysiological parameters were measured using TMS. Participants were seated in an armchair with their forearms pronated, fully relaxed, and supported by armrests. TMS was administered with a magnetic stimulator (NS3000, YIRUIDE, Wuhan, China) connected to a figure-of-eight coil, which was positioned tangentially to the scalp over the contralesional M1 hand area. The coil handle was positioned posterolaterally at approximately 45 degrees from the midsagittal line to direct the induced current in the posteroanterior direction of the brain. The motor hotspot was determined by systematically mapping the presumed hand area to locate the site that consistently elicited the largest and most reproducible MEPs from the contralesional first dorsal interosseous muscle at rest. This site was selected due to its consistent production of reliable MEPs for hotspot localization and paired-pulse TMS in this cohort and was maintained for all subsequent testing. Neuronavigation was not used in this study.

The resting motor threshold (RMT) was determined at the identified hotspot and was defined as the minimum stimulus intensity required to evoke a recognizable MEP (amplitude > 0.05 mV) in at least 5 of 10 consecutive stimulations when the target muscle was completely relaxed.

The baseline TMS intensity for each participant was determined as the stimulator output that generated an average MEP amplitude closest to 1 mV, within the range of 0.5–1 mV, across 10 trials with the target muscle fully relaxed. Baseline MEP was evoked 20 times at a frequency of 0.2 Hz using baseline TMS intensity, which was then averaged.

Short- and long-ICI were subsequently assessed in different sessions. For short-ICI, a conditioning stimulus set at 70%RMT was applied, followed by a test stimulus at the baseline TMS intensity, with an interstimulus interval of 2 ms [18]. For long-ICI, a conditioning stimulus set at 120%RMT was applied, followed by a test stimulus at the baseline TMS intensity, with an interstimulus interval of 150 ms [37]. Short- and long-ICI were calculated as a percentage ratio of the MEP amplitude induced by a paired-pulse stimulus to the baseline MEP (i.e., ICI (%) = MEP amplitude induced by paired pulses/baseline MEP × 100%). Thus, values < 100% indicate inhibition, whereas values ≥ 100% indicate reduced inhibition (disinhibition) or net facilitation relative to the unconditioned response. Both short- and long-ICI were collected 20 times at a frequency of 0.2 Hz and averaged, respectively.

### 2.4. Measurement Processing

**Figure 2:**
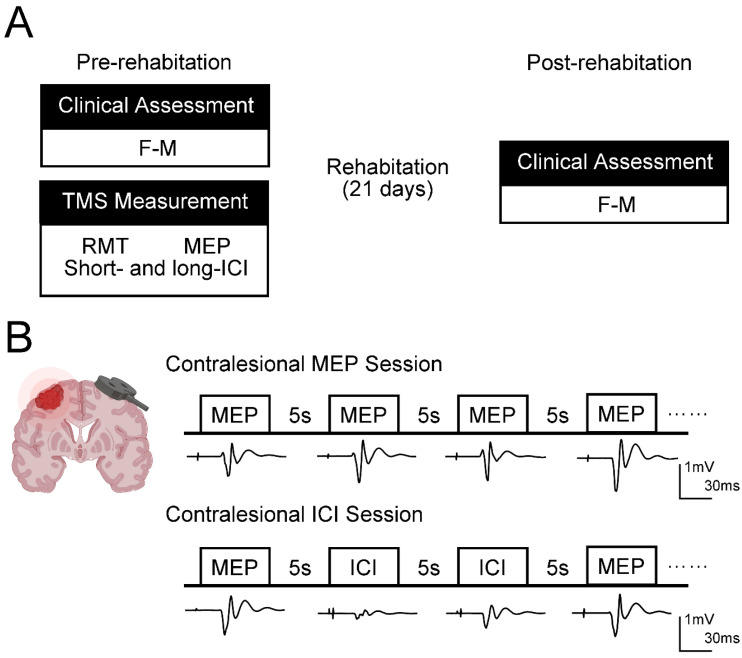
Experimental design. (**A**) Experimental protocol. Patients were measured for F-M, RMT, MEP, short- and long-ICI before rehabilitation. F-M was always measured first to screen patients for inclusion in this study. RMT, MEP, and ICI were measured on another day after the F-M. All the TMS measurements were completed on the same day. (**B**) TMS measurement. TMS was used to evaluate the neurophysiological parameters on the contralesional (unaffected) side. The coil was placed on the M1 projection area of the contralesional first dorsal interosseous muscle. RMT and MEP were always measured first to determine the conditioning stimulus and test stimulus. The measurement order of short- and long-ICI was randomized. Given the excessively long overall testing duration and the absence of neuro-navigation guidance, to avoid movement of the coil and to preclude the potential changes in the corticospinal excitability of patients during ICI measurements, a single-pulse stimulus with the baseline TMS intensity was followed by two paired-pulse stimuli. F-M, Fugl–Meyer Assessment; RMT, resting motor threshold; MEP, motor-evoked potential; ICI, intracortical inhibition; TMS, transcranial magnetic stimulation.

Figure 2 illustrates the measurement process. The F-M, RMT, MEP, short-ICI, and long-ICI were measured before rehabilitation. The F-M was always measured first to screen patients for inclusion in this study. Contralesional RMT, MEP, and ICI were measured on the day after F-M. All the TMS measurements were performed on the same day. Although RMT and MEP were always measured first to determine the conditioning and test stimuli for the ICI sessions, the measurement order of short- and long-ICI was randomized. Furthermore, given the excessively long overall testing duration and the absence of neuro-navigation guidance, a single-pulse stimulus with the baseline TMS intensity was followed by two paired-pulse stimuli to avoid movement of the coil and preclude potential changes in the corticospinal excitability of patients during ICI measurements. Thirty stimuli were recorded in each session of both short- and long-ICI (10 trials of MEPs and 20 trials of ICI).

The patients were re-evaluated using the F-M after 21 days of comprehensive rehabilitation. The comprehensive rehabilitation program, designed by the attending physician based on the clinical condition of each patient, included individualized inpatient physical and occupational therapy. Each form of therapy was administered daily for 30–60 min. Rehabilitation intensity was not entered as a covariate in the analyses because treatment content and intensity were individualized and were not prospectively standardized in a protocolized manner, which should be considered when interpreting the results.

### 2.5. Electrophysiological Recordings and MEP Analysis

Surface electromyography (EMG) recordings were obtained using Ag–AgCl electrodes from the first dorsal interosseous muscle on the contralesional side. The signals were amplified, bandpass filtered between 20 Hz and 2 kHz, and digitized at 5000 Hz using the acquisition module integrated with the TMS system (YIRUIDE NS3000, China). EMG signals were time-locked to the TMS pulse (t = 0), saved in 100-ms epochs, and stored on a computer for offline analysis.

The acquired raw EMG signals were analyzed offline using MATLAB version R2024a (MathWorks Inc., Natick, MA, USA) with custom scripts. Due to equipment limitations, we were unable to directly obtain the resting-state EMG signal immediately before each TMS pulse. Therefore, based on the approximate range of MEP latency, we calculated the EMG signal within the 5–20 ms time window post-trigger as the background muscle activity. Any trial with background activity > 50 µV was excluded to ensure that the MEPs were elicited under relaxed muscle conditions. Additionally, we manually inspected all trials and removed those with obvious artifacts caused by TMS coil clicks or accidental participant movements.

Considering the significantly prolonged MEP latency on the unaffected side in patients with stroke, the MEP amplitude was measured as a peak-to-peak value within the 20–60 ms post-stimulus time window. The average MEP amplitude across all valid trials for each experimental condition was used as the MEP amplitude for that stimulation condition in subsequent analysis.

### 2.6. Statistics

Values are expressed as mean ± standard error. The coefficients of variation for neurophysiological parameters (i.e., MEP, short- and long-ICI) were calculated to assess the cortical stability of each patient.

The Shapiro–Wilk test was used to verify whether all indicators (rehabilitation effect, RMT, and the amplitudes and coefficients of variation of MEP and ICI) conformed to a normal distribution. If the rehabilitation effect followed a normal distribution, a two-tailed paired sample *t*-test was conducted on the total scores and 10 sub-items of the F-M between baseline (admission) and post-rehabilitation (after 21 days) to identify the sub-items with significant rehabilitation improvements. The Wilcoxon paired signed-rank test was used instead of the paired sample *t*-test if the data did not follow a normal distribution.

If the neurophysiological parameters followed a normal distribution, one-way repeated-measures analysis of variance (rmANOVA) was used to test for differences in amplitude and coefficient of variation among MEP trials within three single-pulse measurements, i.e., MEP sessions, short- and long-ICI sessions. The Friedman test was used instead of the rmANOVA if the data did not follow a normal distribution, to prove that the MEP amplitude of the test stimulation alone did not show significant fluctuations during the ICI measurement process, and no shifts in the position of the coil were observed. A one-sample *t*-test was used to verify whether short- and long-ICI were <100%; if so, the intracortical circuit inhibition was considered significant. The Wilcoxon paired signed-rank test was used if the data were not normally distributed. A two-tailed independent samples *t*-test was used to test the differences in RMT, MEP, and ICI between the improvement and non-improvement groups. The Mann–Whitney U test was used instead of the independent samples *t*-test if the data did not follow a normal distribution.

A prediction model was established with the rehabilitation effects (rehabilitation_t__otal_, rehabilitation_proximal_, rehabilitation_distal_, and rehabilitation_whole_) as response variables and neurophysiological parameters (RMT, MEP, short-ICI, long-ICI, coefficient of variation of MEP, coefficient of variation of short-ICI, and coefficient of variation of long-ICI) as predictors. If the rehabilitation effects did not follow a normal distribution, considering that all rehabilitation effects were non-negative integers leading to a possibility of them being zero, the rehabilitation effects were square-root transformed before subsequent analysis to simplify the modeling process.

Simple linear regression and logistic regression were performed for each predictor to evaluate its predictive ability for rehabilitation effects (changes in score) and status (improvement or non-improvement). Furthermore, a multiple prediction model was constructed using all the predictors. However, as the neurophysiological parameters included in this study varied in units and numerical ranges, we transformed all neurophysiological parameters to Z-score before multivariate model analysis to standardize the measurement scales of different variables. The response variables were divided into two parts. In one part, the dependent variable was a continuous variable, using the rehabilitation scores (after square-root transformation if non-normally distributed) of each patient directly. In addition, we reported models with untransformed rehabilitation scores as the dependent variable for sensitivity analysis. In the other part, the dependent variable was a binary variable. The patients were divided into two groups based on their raw rehabilitation scores (i.e., without transformation): an improvement group and a non-improvement group. The improvement group was coded as 1, and the non-improvement group was coded as 0. A generalized linear model with a linear distribution was used to predict the rehabilitation effects (changes in score). Despite Z-score standardization, the distribution of some independent variables might not adequately satisfy the normality assumption. In this regard, the generalized linear model framework offers greater flexibility in error distribution assumptions, making it more robust than standard multiple linear regression. Binary logistic regression was used to predict rehabilitation status (improvement or non-improvement). This method effectively distinguished “responders (patients with improvement)” from “non-responders (patients without improvement)” rather than predicting the magnitude of continuous score changes. Receiver operating characteristic curve analysis was performed on the predicted values of the binary logistic regression to assess the ability of the neurophysiological parameters to predict rehabilitation effects in terms of sensitivity, specificity, and area under the curve (AUC) with a 95% confidence interval. We performed 5-fold cross-validation and Bootstrap resampling validation for each statistically significant generalized linear and binary logistic regression model and evaluated model calibration using the Hosmer–Lemeshow goodness-of-fit test to assess the generalization ability of the model. Finally, in models that demonstrated statistical significance, we will include baseline impairment severity and disease duration—both standardized via Z-score transformation—as covariates. This step will allow us to evaluate whether the neurophysiological parameters provide added predictive value beyond conventional clinical predictors.

Statistical significance was set at *p* = 0.05, except for the rehabilitation effect analyses, where a Bonferroni-corrected threshold of *p* < 0.005 (i.e., 0.05/10 sub-items) was applied. SPSS version 27.0 (IBM, Armonk, NY, USA) and MATLAB version R2024a (MathWorks Inc., Natick, MA, USA) were used for statistical and regression analyses.

## 3. Results

### 3.1. Rehabilitation Effect

After 21 days of rehabilitation, only one of the 40 patients failed to show an increase in the total F-M score (change in score = 0). None of the patients experienced deterioration in their total F-M scores after rehabilitation (change in score < 0). The Shapiro–Wilk test showed that none of the rehabilitation effects conformed to a normal distribution (Supplementary Table S2). According to the minimum clinical significance, a total of 22 patients had an increase of ≥6 points in their total F-M scores and were regarded as the “improvement” group, while the other 18 patients were considered the “non-improvement” group.

**Figure 3:**
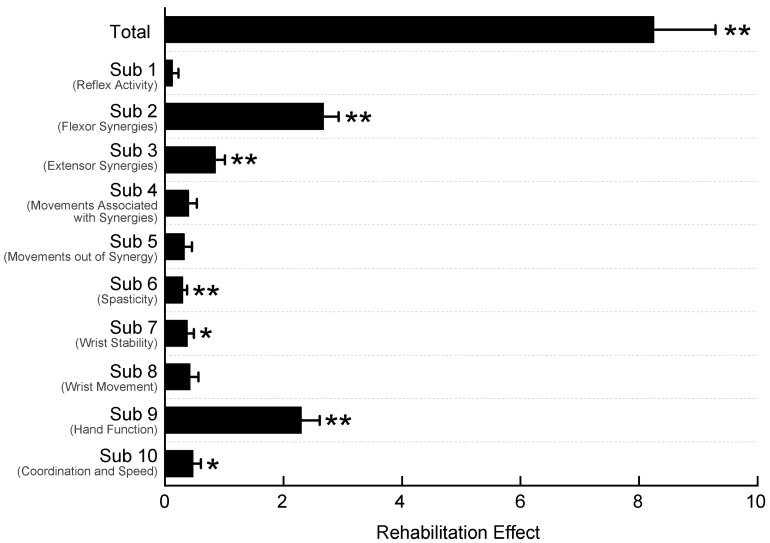
Rehabilitation effect on the patients. Data are presented as mean ± standard error. The ordinate represented the total F-M score and each sub-item from top to bottom. The abscissa represents the level of rehabilitation effect. Rehabilitation effect = F-M_post-rehabilitation_ − F-M_baseline_. None of the patients experienced a deterioration in the total F-M score after rehabilitation (i.e., change in score < 0). * *p* < 0.05, ** *p* < 0.01. F-M, Fugl–Meyer Assessment. Sub 1, 2, …, 10 represent the scores of the first, second … and tenth sub-items for Fugl–Meyer Assessment. Each sub-item title corresponds to the terminology defined in the FM [38].

Figure 3 shows the rehabilitation effects for all patients. Compared with baseline, the total F-M score significantly increased (Wilcoxon paired signed-rank test, Z = −5.45, *p* < 0.001, r = 0.86). Further analysis showed that the scores of the second, third, sixth, seventh, ninth, and tenth sub-items were also significant (Bonferroni-corrected threshold of *p* < 0.005). The statistical results for all sub-items are shown in Supplementary Table S3.

### 3.2. Neurophysiological Parameters

No discomfort or side effects were reported by any patient during TMS measurement. All the participants were assessed for MEP and short-ICI. However, due to factors such as the physical condition of some patients, only 34 patients were assessed for long-ICI. Therefore, participants with missing long-ICI data were excluded from statistical and modeling analyses involving long-ICI (final *n* = 34) to ensure that all analyses for a given model were based on the same subset of participants. All other analyses that did not involve long-ICI utilized the complete cohort (*n* = 40).

Table 1 shows the neurophysiological parameters of the contralesional side. None of the TMS measurements conformed to a normal distribution, except for the coefficient of variation value of short-ICI. The Shapiro–Wilk test results for each TMS measurement are shown in Supplementary Table S4. The comparison among MEP trials within three single-pulse measurements showed no significant differences in terms of both amplitude and coefficient of variation value (Friedman test; amplitude: χ^2^ = 0.41, df = 2, *p* = 0.814, W = 0.01; coefficient of variation: χ^2^ = 2.88, df = 2, *p* = 0.237, W = 0.04). Comparison of MEP trials across three single-pulse measurements revealed no significant differences in amplitude or coefficient of variation. These findings indicate that corticospinal excitability in patients with stroke remained stable throughout MEP and ICI assessments. No significant differences were observed between the unconditioned MEP and the conditioned paired-pulse responses (Wilcoxon paired signed-rank tests. MEP vs. short-ICI, Z = −0.54, *p* = 0.591, r = 0.09; MEP vs. long-ICI, Z = −0.11, *p* = 0.912, r = 0.02). There were no significant differences in RMT, MEP, short- and long-ICI between the improvement and non-improvement groups, either in terms of amplitude or coefficient of variation (Figure S1, Mann–Whitney U test; RMT: U = 136, Z = −1.50, *p* = 0.140, r = 0.24. Amplitude: MEP, U = 158, Z = −1.09, *p* = 0.277, r = 0.17; short-ICI: U = 164, Z = −0.92, *p* = 0.355, r = 0.15; long-ICI: U = 125, Z = −0.53, *p* = 0.600, r = 0.09. Coefficient of variation: MEP, U = 171, Z = −0.73, *p* = 0.463, r = 0.12; short-ICI: U = 150, Z = −1.31, *p* = 0.192, r = 0.21; long-ICI: U = 92, Z = −1.68, *p* = 0.093, r = 0.29).

### 3.3. Neurophysiological Parameters and Rehabilitation Effect

We explored the relationship between individual neurophysiological parameters and the rehabilitation effects. The rehabilitation effects were evaluated in two parts: the change in score and the improvement status. We investigated not only the change in the total F-M score but also the changes in the scores of the proximal, distal, and whole limb (rehabilitation_total_, rehabilitation_proximal_, rehabilitation_distal_, and rehabilitation_whole_). There was no significant univariate linear relationship between any of the seven neurophysiological parameters and any of the rehabilitation effects. Univariate models using long-ICI-related predictors were based on the available cases for that measure (*n* = 34). Supplementary Tables S5 and S6, respectively, show the univariate regression results for each neurophysiological parameter with the rehabilitation effects. Supplementary Tables S7–S9 further present the results of three separate univariate linear regression analyses performed as part of the sensitivity analyses: (1) between Z-scored neurophysiological parameters and the original rehabilitation effect scores; (2) between original neurophysiological parameters and the original rehabilitation effect scores; (3) between original neurophysiological parameters and the rehabilitation status (improvement/non-improvement). The results of the sensitivity analysis are consistent with those from the original model. The sensitivity analysis yielded consistent findings, showing no significant linear relationship between the seven neurophysiological parameters and any rehabilitation effects.

A generalized linear model based on a linear distribution was used to explore the relationship between square-root transformed rehabilitation effects and neurophysiological parameters. Multivariable models that included all predictors were performed as complete-case analyses (*n* = 34) because long-ICI data were missing in six participants. The results showed that there was no significant relationship between the seven neurophysiological parameters and all rehabilitation effects (rehabilitation_total_: χ^2^_LG_ = 5.32, df = 7, *p* = 0.621; rehabilitation_proximal_: χ^2^_LG_ = 4.80, df = 7, *p* = 0.685; rehabilitation_distal_: χ^2^_LG_ = 5.63, df = 7, *p* = 0.584; rehabilitation_whole_: χ^2^_LG_ = 4.00, df = 7, *p* = 0.781). Supplementary Tables S10 and S11 further present the results of two generalized linear models performed as part of the sensitivity analyses: (1) between Z-scored neurophysiological parameters and the original rehabilitation effect scores and (2) between original neurophysiological parameters and the original rehabilitation effect scores. The sensitivity analysis yielded consistent findings, showing no significant relationship between the seven neurophysiological parameters and all rehabilitation effects.

**Figure 4:**
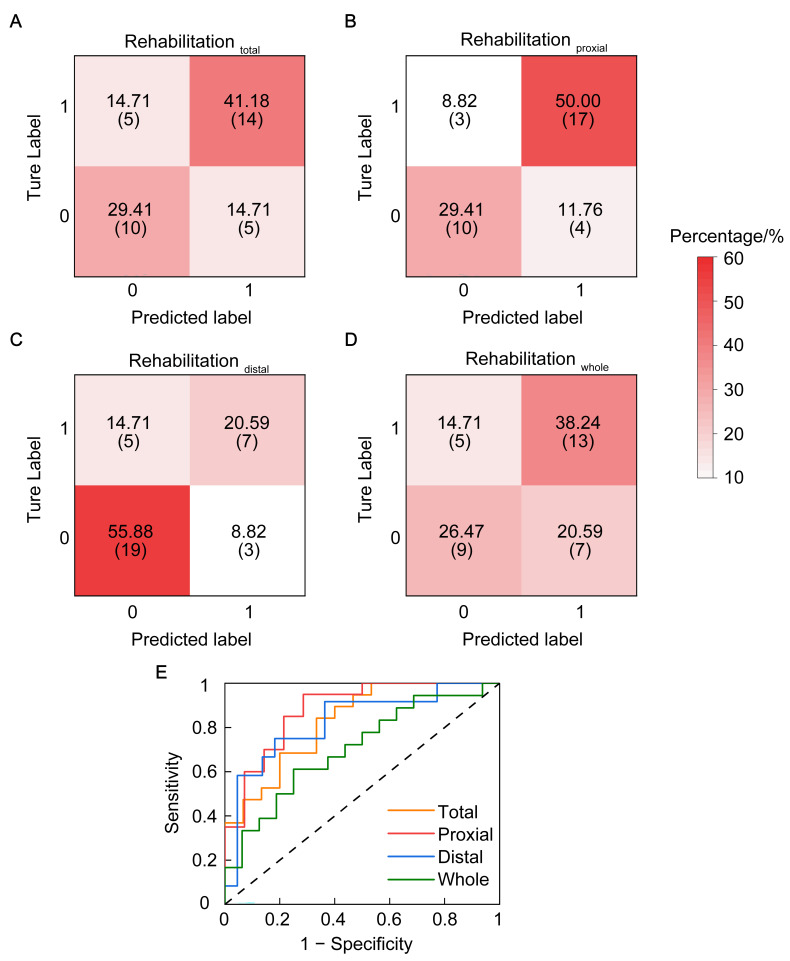
Relationship between rehabilitation status (improvement/non-improvement) and all neurophysiological parameters. (**A**–**D**) Heatmap of the confusion matrix for the results of binary logistic regression. The predictors were all neurological parameters. The predicted values from (**A**–**D**) were the rehabilitation effect in the total F-M score, the proximal limb function (the second, third, fourth and fifth sub-items of upper-limb F-M score), the distal limb function (the seventh, eighth and ninth sub-items of upper-limb F-M score), and the whole limb motor function (the first, sixth and tenth sub-items of upper-limb F-M score). Patients were divided into improvement and non-improvement groups according to the minimal clinical difference; 1 represented improvement, and 0 represented non-improvement. The vertical axis represents the true label, which indicates the actual counts and proportions of classes 1 and 0 in the real environment. The horizontal axis represents the predicted label, which shows the distribution of classes 1 and 0 as determined by the model predictions. The values in each cell were presented as percentages (actual number of patients). Darker colors in the cells indicated a higher proportion. (**E**) Receiver operating characteristic curve of each model. Orange, red, blue, and green lines represent the rehabilitation effect in the total F-M score, proximal limb function, distal limb function, and integrated function predicted by the model. The black dotted line is the reference line. The vertical axis represented sensitivity, and the horizontal axis represented 1 − specificity. F-M, Fugl–Meyer assessment.

Furthermore, we used binary logistic regression to examine the relationship between neurophysiological parameters and rehabilitation status (improvement/non-improvement) within the same complete-case subset (*n* = 34). As shown in Figure 4, a significant relationship was noted between neurophysiological parameters and the improvement effect of proximal limbs (rehabilitation_proximal_) (χ^2^_LG_ = 28.79, *p* = 0.016, AUC = 0.88, R^2^ = 0.54, AIC = 44.79, BIC = 57.00). Table 2 presents the parameter estimates for each factor in this model. As shown in Table 2, only short-ICI was a significant factor for rehabilitation_proximal_. The Hosmer–Lemeshow goodness-of-fit test indicated no significant lack of fit (χ^2^ = 3.85, df = 8, *p* = 0.871), demonstrating that the predicted probabilities from the model were consistent with the observed proportions of motor recovery in our cohort.

**Figure 5:**
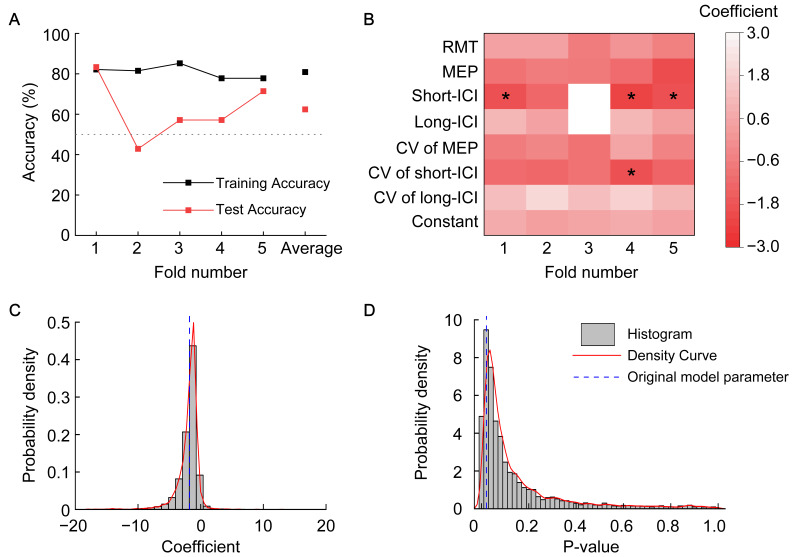
Performance validation of the proximal recovery prediction model. (**A**) Comparison of overall accuracy in 5-fold cross-validation. The black and red lines represent the accuracies of the training and test sets for each fold, respectively, and are averaged over all five folds. The vertical axis indicates accuracy, and the horizontal axis represents the fold number. (**B**) Coefficients and significance of each predictor in 5-fold cross-validation. The vertical axis lists, from top to bottom, the seven predictors and the constant term. The horizontal axis denotes each fold. Values in each cell are presented as coefficients. Factors that reached significance in the corresponding fold are marked with * (*p* < 0.05). (**C**,**D**) Distribution plots of bootstrap resampling results (*n* = 5000) for Short-ICI, the significant predictor in the original model. The vertical axis shows probability density, calculated as frequency/(total sample count × bin width). The horizontal axis represents the coefficient (**C**) and the *p*-value (**D**). The red curve indicates the probability density curve. Blue dashed lines mark the position of the coefficient (−1.782) and *p*-value (0.021) of Short-ICI from the original model. RMT, resting motor threshold; MEP, motor-evoked potential; ICI, intracortical inhibition; CV, coefficient of variation.

To assess model robustness, we further conducted five-fold cross-validation and Bootstrap resampling analysis. As shown in Figure 5A,B, the five-fold cross-validation indicated a potential risk of overfitting in the model (mean training accuracy 0.804; mean test accuracy 0.62; overfitting gap 0.19). However, the significant predictor short-ICI identified in the original model remained significant in three out of the five folds. To further examine the stability of short-ICI, bootstrap resampling analysis was performed. As shown in Figure 5C,D, the original coefficient and *p*-values for the significant variable short-ICI both exhibited a right-skewed distribution and clustered within the modal region, indicating a certain degree of statistical robustness. Supplementary Figures S2 and S3 present the Bootstrap resampling analysis results for other non-significant variables in the model, providing complementary information for robustness assessment.

No significant relationship was noted between the neurophysiological parameter and rehabilitation total (χ^2^_LG_ = 32.67, *p* = 0.051, AUC = 0.83, R^2^ = 0.45, AIC = 48.67, BIC = 60.88), rehabilitation distal (χ^2^_LG_ = 32.89, *p* = 0.128, AUC = 0.83, R^2^ = 0.39, AIC = 48.89, BIC = 61.10), or rehabilitation whole (χ^2^_LG_ = 42.24, *p* = 0.69, AUC = 0.71, R^2^ = 0.18, AIC = 58.24, BIC = 70.46). Supplementary Table S12 further presents the results of binary logistic regression performed as part of the sensitivity analyses between original neurophysiological parameters and the rehabilitation status (improvement/non-improvement). The results of the sensitivity analysis are consistent with those of the original model in predicting proximal limb rehabilitation. Furthermore, the sensitivity analysis revealed the significance of neurological parameters in predicting overall recovery (rehabilitation_total_, *p* = 0.038).

### 3.4. Incremental Value of Neurophysiological Parameters Beyond Clinical Predictors

To evaluate whether TMS-derived neurophysiological parameters could provide predictive information beyond standard clinical measures, we conducted an incremental value analysis. First, building upon the original, statistically significant proximal recovery prediction model, we incorporated baseline impairment severity (proximal F-M score at admission) and disease duration as clinical covariates to construct an enhanced predictive model. While this augmented model demonstrated excellent discriminative ability (χ^2^_LG_ = 26.08; *p* = 0.002; AUC = 0.95) and a high degree of explained variance (R^2^ = 0.72), the Hosmer–Lemeshow goodness-of-fit test indicated poor calibration (*p* < 0.001). Five-fold cross-validation indicated a more pronounced risk of overfitting in this augmented model compared to the original predictive model (mean training accuracy: 0.94; mean test accuracy: 0.70; overfitting gap: 0.24), primarily attributable to the limited sample size of this study (*n* = 34).

Thus, we subsequently developed a simplified model. This model featured short-ICI—the predictor identified as making a significant contribution in the original Proximal Recovery Prediction Model (Table 2)—as the core predictor, while adjusting for the same clinical covariates (baseline impairment severity and disease duration). This simplified model was compared against a benchmark logistic regression model containing only baseline impairment severity and disease duration. As shown in Table 3, both models were statistically significant, yet the simplified model achieved a higher Nagelkerke R^2^ value. This finding indicates that short-ICI provides effective incremental predictive information for post-stroke motor recovery prognosis, beyond that offered by standard clinical predictors.

## 4. Discussion

We included 40 stroke patients in the subacute phase (disease course > 14 days to 6 months). All patients were diagnosed with moderate-to-severe upper-limb motor dysfunction, with an average F-M score of only 11.65 ± 2.23 points for the upper limb. None of the patients had an ipsilesional MEP response, indicating a loss of corticospinal tract function. By collecting RMT, MEP, and ICI data on the contralesional side before rehabilitation, we aimed to explore the association between pre-rehabilitation neurophysiological parameters and subsequent rehabilitation effects. The rehabilitation effect was evaluated based on changes in the upper-limb part of the F-M before and after 21 days of rehabilitation. Significant improvements were observed in the total score and in multiple sub-items. We further refined the rehabilitation effect into three subcategories according to the F-M test content: proximal, distal, and whole functions.

To ensure consistency of corticospinal tract excitability in the patient cohort, a single-pulse stimulus was interspersed during the ICI session. Results of the analysis revealed no significant differences in the MEP amplitudes evoked by the interspersed single-pulse stimuli across the baseline, short-ICI, and long-ICI sessions, indicating no deviation of the stimulation target and stable corticospinal tract excitability during TMS measurement. The paired-pulse response was expressed as conditioned/unconditioned MEP × 100; therefore, values > 100% should be interpreted as reduced ICI (disinhibition) or net facilitation on the contralesional side rather than inhibition in the strict physiological sense. Under these conditions, we found that the average ICI (both short- and long-ICI) exceeded 100%. This suggests a generalized loss of ICI capacity in the contralesional hemisphere in patients with corticospinal tract damage and aligns with the altered excitatory–inhibitory balance characteristic of stroke [27]. This disinhibition of M1 intracortical circuits—observed under stable contralesional corticospinal excitability—could reflect the maintenance of a relatively “optimized” rather than “excessively reduced” level of GABAergic inhibition in the contralesional hemisphere. This alteration might represent an adaptive process within the motor inhibitory network after stroke [30]. Such adaptive remodeling of inhibition might contribute to establishing a new excitatory–inhibitory balance in the contralesional hemisphere, which could subsequently support compensatory functions. However, methodological considerations such as the absence of neuronavigation and the inherent variability of paired-pulse measures must also be considered.

Patients were grouped according to the minimal clinically important difference in the F-M [36], resulting in an improved group (*n* = 22) and a non-improved group (*n* = 18). No significant differences in neurophysiological parameters were found between these groups, nor was any single parameter significantly associated with score changes or improvement status. This suggests that for patients without ipsilesional MEP, a single neurophysiological parameter of the contralesional side has a poor predictive ability for motor function recovery after stroke rehabilitation, which is consistent with the results of previous studies [39].

Further analysis revealed that although these contralesional neurophysiological measures showed limited predictive value for overall rehabilitation outcome, they exhibited an exploratory association with improvements in proximal limb motor function during the early rehabilitation phase. Robustness assessments, including cross-validation, indicated a potential risk of overfitting (Figure 5A). This risk primarily stems from the limited sample size (*n* = 34) and model over-adaptation, which constrain the generalizability of the findings. Although 5-fold cross-validation and bootstrap analysis further demonstrated that the key predictor (short-ICI) retained a certain degree of statistical stability (Figure 5B–D), these associative results should be interpreted with caution. Other possible explanations for the observed predictive pattern include the loss of information owing to endpoint dichotomization and dependence on the specific cutoff values chosen. Although a biologically meaningful “threshold effect” in recovery remains a possible interpretation, it must be regarded as speculative given the current analytical limitations. Future studies should adopt more refined modeling methods to explore how neural parameters can predict changes in continuous scores.

Within the predictive model, short-ICI emerged as a parameter of particular interest. This exploratory finding suggests that neurophysiological measures derived from the contralesional hand motor area may be tentatively linked to the responsiveness of proximal motor function during early rehabilitation, a hypothesis that requires confirmation in future studies. Furthermore, incremental analysis indicated that short-ICI could provide independent predictive information for proximal limb recovery beyond conventional clinical measures. However, these results are preliminary, and their potential clinical utility remains speculative.

Notably, short-ICI alone was not significantly correlated with proximal motor recovery. This finding is consistent with a recent study that found no direct association between the F-M and ICI [40]. This further suggests that in patients with corticospinal tract injury, motor recovery may depend on the integrated function of the contralesional motor cortex—encompassing its intracortical inhibition, cortical excitability, and the integrity of descending pathways. Such an integrated function could reflect compensatory recruitment of a cortico-reticulo-spinal route for proximal control, though this interpretation remains hypothetical and indirect [41]. In humans, the reticulospinal system shows increased excitability after severe corticospinal tract damage and has been repeatedly linked to the recovery of gross strength and synergy-based proximal movements, rather than fine motor selectivity [34]. In contrast, individuated finger movements depend disproportionately on primate-specific cortico-motoneuronal projections within the corticospinal tract. Damage to these projections seems to limit the restoration of distal dexterity, even when proximal stabilization improves [35]. Neuroimaging further shows task- and stage-dependent recruitment of the contralesional M1 during the recovery of reaching and shoulder–elbow control, which is consistent with a supportive, though not universally beneficial, role of this network [42]. Furthermore, ipsilesional MEPs are more readily elicited in the proximal muscles and can be facilitated by startle-based brainstem conditioning, which is consistent with a possible reticulospinal system-mediated contribution [43]. However, a previous study indicated that ipsilesional corticospinal tract integrity is the primary determinant of upper-limb skill [44], underscoring that neurophysiological parameters from the contralesional hemisphere should be interpreted as biomarkers of compensatory recruitment rather than causal drivers. Accordingly, the potential reticulospinal mechanism suggested here should be viewed as an indirect, hypothesis-generating interpretation, since this study did not directly measure reticulospinal tract activity or structural connectivity.

The findings of this study may be further understood in the context of bilateral brain activation patterns after stroke. Pundik et al. reported that recovery of shoulder–elbow reach may involve recruitment of bilateral primary motor regions and contralesional motor-sensory areas, particularly in patients with greater baseline impairment, whereas less impaired patients may show a more focused activation pattern [42]. This suggests that proximal recovery can engage bilateral and contralesional compensatory networks. However, this framework should not be generalized to distal hand recovery. Previous studies have shown that, during hand-related tasks, greater CST disruption is associated with a shift from ipsilesional primary motor recruitment toward contralesional and bilateral premotor recruitment, but hand performance remains strongly influenced by ipsilesional CST integrity [45,46]. In particular, precision grip, finger individuation, long-term hand function, and distal upper-extremity improvement after rehabilitation all appear to remain closely associated with residual CST integrity [47]. Accordingly, the association of contralesional neurophysiological markers with proximal rather than distal recovery in our study might be better interpreted as evidence of compensatory network recruitment for proximal control, rather than evidence of distal recovery becoming independent of the damaged ipsilesional CST.

The classification of F-M into “proximal, distal, and whole-limb” categories in this study is fundamentally an anatomy-based operational framework and not an independently validated, standardized alternative scoring system. The primary positive finding of this analysis specifically relies on the “proximal” grouping. As a result, the observed association may be partially shaped by the specific operational definition employed. Further interpretation of these findings should remain strictly within the confines of the anatomical framework applied here. Future studies are needed to further validate and clarify the clinical relevance of these anatomical classifications.

This study had some limitations. First, the small sample size and absence of long-ICI data for some patients carry the risk of model overfitting and limit the generalizability of the findings. It must be explicitly noted that this restricts the statistical power and broader applicability of the results, which is a key methodological limitation. Nevertheless, we exploratorily identified potential neurophysiological candidate predictors derived from contralateral ICI measures associated with improvement in proximal limb function on the affected side. These findings provide preliminary clues for further research into the role of ICI in stroke rehabilitation; nonetheless, rigorous external validation and calibration in larger prospective cohorts are warranted. Second, corticospinal tract damage was inferred neurophysiologically from the absence of ipsilesional MEPs because quantitative lesion characteristics, lesion volume, and diffusion-based CST measures were not available. Third, the variability in rehabilitation protocols and intensity, as well as medication use, was not prospectively standardized or controlled for as covariates. This lack of standardization is a recognized limitation that may have introduced confounding effects on cortical excitability and recovery outcomes, thereby affecting the robustness of our conclusions. The sources of heterogeneity in rehabilitation—such as differences in session frequency, therapeutic techniques, and individualized adjustments—could influence both neurophysiological measures and functional recovery. Since the incremental analysis conducted by additionally incorporating known key clinical factors further highlights the importance of these critical clinical confounders in predictive models, future studies may benefit from prospectively controlling or stratifying rehabilitation parameters to minimize such variability. Fourth, although single-pulse MEPs remained stable across blocks, the lack of neuronavigation likely reduced the accuracy and reproducibility of paired-pulse TMS measurements, representing a substantial technical limitation in our neurophysiological assessments. This study focused only on typical TMS indicators (RMT, MEP, and ICI) and applied data transformation to make them suitable for regression modeling. The choice of single-modality predictors, data transformation methods, and regression models may all impact the prediction accuracy. Moreover, this study lacks analysis of neurophysiological parameters during the post-rehabilitation evaluation stage. Future research should incorporate key clinical factors to more comprehensively explore the relationships between various biomarkers and rehabilitation outcomes. Finally, this study only assessed rehabilitation effects within 21 days, which primarily captures responsiveness in the early phase and does not represent long-term prognosis.

Therefore, future studies should extend the follow-up period to further clarify the impact of rehabilitation interventions on long-term motor outcomes. Additionally, rigorous external validation in larger-scale, multi-center prospective independent cohorts is needed, integrating a wider range of biomarkers to build comprehensive and clinically applicable models. This requires incorporating multiple indicators across broader patient populations, including expanded TMS measures such as MEP latency and the silent period, neuroimaging markers [16], and cortical connectivity assessments from combined electroencephalography-TMS techniques [48]. Furthermore, more key clinical factors—including lesion characteristics and treatment intensity—should be included as covariates to accurately predict post-stroke motor recovery. Proximal muscles like the deltoid or biceps should also be included in TMS assessments [34,40] to address the mismatch between the assessment site (first dorsal interosseous muscle was considered the hotspot in this study) and clinically observed features, and multimodal mapping techniques could be employed to more directly test this interpretation.

## 5. Conclusions

Patients with stroke with moderate-to-severe motor dysfunction and damaged corticospinal tract function on the ipsilesional side exhibited poor predictability for motor function recovery after stroke rehabilitation when assessed using a single neurophysiological parameter from the contralesional side. However, our findings suggest a preliminary and exploratory association between a multi-parameter model integrating TMS indicators from the contralesional side and the potential to improve prediction of proximal limb motor function recovery. These results highlight the possible incremental value of a multi-parameter approach over single measures, particularly with short-ICI showing promise for further clinical investigation. Thus, these findings generate the hypothesis that contralesional neurophysiological measures could help characterize compensatory processes related to proximal recovery, which might indirectly align with the postulated compensatory role of the cortico-reticulo-spinal pathway. However, the possible involvement of the cortico-reticulo-spinal pathway remains speculative rather than causal, as this study did not directly assess activity or structural connectivity of this pathway. Future larger-scale prospective studies are essential to externally validate these exploratory findings, to clarify their true predictive utility, and to confirm the clinical relevance and generalizability of these observed, albeit preliminary, associations before any clinical predictive use can be considered. This study offers exploratory perspectives and directions for research in the field of stroke rehabilitation.

## Figures and Tables

**Table 1:** Values of neurophysiological parameters for TMS measurements.

	Value
RMT (%MSO)	38.43 ± 1.90
Baseline TMS intensity (%MSO)	52.63 ± 2.76
MEP amplitude (mV)	0.60 ± 0.07
Short-ICI (%)	140.56 ± 24.67
Long-ICI (%)	131.23 ± 26.24
Coefficient of variation of MEP	0.60 ± 0.04
Coefficient of variation of short-ICI	0.57 ± 0.03
Coefficient of variation of long-ICI	0.52 ± 0.04

Data are presented as mean ± standard error. TMS, transcranial magnetic stimulation; RMT, resting motor threshold; MSO, maximum stimulus output; MEP, motor-evoked potential; ICI, intracortical inhibition.

**Table 2:** Parameter estimates for the proximal recovery prediction model.

	Coefficient	Wald	OR (95%CIs)	*p*
RMT	0.02	0.002	1.02 (0.37, 2.81)	0.965
MEP amplitude	−0.95	1.96	0.39 (0.10, 1.46)	0.162
Short-ICI	−1.78	5.33	0.17 (0.04, 0.76)	0.021 *
Long-ICI	0.85	1.31	2.35 (0.54, 10.14)	0.253
Coefficient of variation of MEP	−0.34	0.29	0.72 (0.21, 2.45)	0.593
Coefficient of variation of short-ICI	−1.22	2.47	0.29 (0.06, 1.35)	0.116
Coefficient of variation of long-ICI	1.21	2.83	3.37 (0.82, 13.84)	0.092
Constant	0.55	1.33	1.74 (0.68, 4.45)	0.250

* *p* < 0.05. CIs, confidence intervals; RMT, resting motor threshold; MEP, motor-evoked potential; ICI, intracortical inhibition.

**Table 3:** Comparison of the simplified model incorporating short-ICI and clinical-predictors-only benchmark model (*n* = 40).

	Simplified Model	Benchmark Model
χ^2^_LG_	12.41	7.02
df	3	2
*p* value	0.006 **	0.030 *
R^2^	0.36	0.22
*p* value of H-L test	0.66	0.73
AUC	0.81	0.71
AIC	50.14	53.53
BIC	56.90	58.60

** *p* < 0.01; * *p* < 0.05. H-L test, Hosmer–Lemeshow goodness-of-fit test.

## Data Availability

The data presented in this study are available on request from the corresponding author due to privacy and ethical restrictions.

## Supplementary Materials

The following supporting information can be downloaded at: https://www.mdpi.com/article/10.3390/brainsci16050505/s1, Table S1. Patient characteristics (*n* = 40). Table S2. The Shapiro–Wilk test result of all rehabilitation effects. Table S3. Difference between pre- and post-rehabilitation score for all sub-items. Table S4. The Shapiro–Wilk test results of all TMS measurements. Table S5. Simple linear regression between Z-scored neurophysiological parameters and square-root transformed rehabilitation score changes. Table S6. Simple logistic regression results between Z-scored neurophysiological parameters and rehabilitation status (improvement/non-improvement). Table S7. Simple linear regression between Z-scored neurophysiological parameters and original rehabilitation score changes. Table S8. Simple linear regression between original neurophysiological parameters and original rehabilitation score changes. Table S9. Simple logistic regression results between original neurophysiological parameters and rehabilitation status (improvement/non-improvement). Table S10. Generalized linear model results between Z-scored neurophysiological parameters and original rehabilitation score changes. Table S11. Generalized linear model results between original neurophysiological parameters and original rehabilitation score changes. Table S12. Binary logistic regression results between original neurophysiological parameters and rehabilitation status (improvement/non-improvement). Figure S1. Between-group comparisons of neurophysiological parameters. Figure S2. Distribution plots of coefficient from bootstrap resampling (*n* = 5000) for the Proximal Recovery Prediction Model. Figure S3. Distribution plots of *p*-value from bootstrap resampling (*n* = 5000) for the Proximal Recovery Prediction Model.
