# Supplementary material for: Neurophysiological Predictors of Proximal Motor Rehabilitation in Stroke Patients with Corticospinal Tract Damage
Source: Brain Sci. 2026 May 8;16(5):505. doi: 10.3390/brainsci16050505 (PMC13204963; doi:10.3390/brainsci16050505)
Supplement: Supplementary file 1 [file brainsci-16-00505-s001.zip › brainsci-4199549-supplementary.pdf]

Supplementary Table S1. Patient characteristics (n = 40).

Patient ID	Gender (M/F)	Age (years)	Disease Duration (months)	Hemisphere (L/R)	Stroke type	F-M $_{total}$	
						Pre	Post
1	M	58	0.5	L	Ischaemic	4	12
2#	M	45	1.0	R	Ischaemic	19	40
3	M	62	1.0	R	Ischaemic	46	58
4	M	59	1.0	R	Ischaemic	6	22
5#	M	58	1.0	R	Ischaemic	7	12
6	M	49	0.5	L	Ischaemic	1	17
7	M	69	0.8	R	Ischaemic	8	17
8	M	53	0.5	R	Haemorrhagic	5	16
9	M	68	0.8	L	Haemorrhagic	5	9
10	M	55	3.0	L	Haemorrhagic	5	10
11	M	63	5.0	R	Haemorrhagic	6	10
12	M	65	1.0	R	Ischaemic	5	8
13#	F	59	1.0	L	Ischaemic	8	13
14	M	42	2.0	R	Haemorrhagic	6	7
15	F	68	6.0	L	Ischaemic	5	7
16	M	45	6.0	L	Haemorrhagic	17	25
17	M	48	1.0	R	Haemorrhagic	5	12
18	M	61	6.0	L	Ischaemic	38	41

19	M	50	5.0	L	Haemorrhagic	13	13
20#	F	65	0.5	R	Ischaemic	5	15
21	M	57	2.0	R	Haemorrhagic	9	35
22	M	51	1.0	R	Ischaemic	5	35
23	F	49	1.0	R	Haemorrhagic	5	9
24	M	60	1.0	R	Ischaemic	5	9
25#	M	36	1.0	L	Haemorrhagic	5	12
26	M	52	6.0	L	Ischaemic	12	19
27	M	66	3.0	L	Ischaemic	0	7
28	M	60	1.0	R	Ischaemic	6	10
29	F	73	6.0	L	Ischaemic	0	4
30	M	40	4.0	R	Ischaemic	28	35
31#	M	56	5.0	R	Ischaemic	4	9
32	M	46	3.0	L	Haemorrhagic	4	12
33	M	59	0.3	L	Haemorrhagic	5	14
34	M	66	2.0	R	Ischaemic	4	12
35	M	65	0.5	R	Ischaemic	4	12
36	M	38	6.0	R	Haemorrhagic	53	56
37	M	62	0.5	L	Ischaemic	6	8
38	M	49	6.0	L	Haemorrhagic	4	9
39	M	67	3.0	L	Ischaemic	50	69
40	M	70	1.0	R	Ischaemic	42	55

Long-ICI data is missing for this patient. A total of 6 patients had missing ICI data.

M, male; F, female; L, left; R, right; F-M $_{total}$, the total score of the upper limb part of the Fugl-Meyer Assessment.

Supplementary Table S2. The Shapiro–Wilk test result of all rehabilitation effects.

	W	df	P
total	0.83	40	<0.001**
sub1	0.20	40	<0.001**
sub2	0.90	40	0.002**
sub3	0.78	40	<0.001**
sub4	0.52	40	<0.001**
sub5	0.44	40	<0.001**
sub6	0.58	40	<0.001**
sub7	0.57	40	<0.001**
sub8	0.55	40	<0.001**
sub9	0.87	40	<0.001**
sub10	0.63	40	<0.001**

Sub 1, 2, …, 10 represent the scores of the first, second … and tenth sub-items for Fugl-Meyer Assessment. **P<0.01.

Supplementary Table S3. Difference between pre– and post–rehabilitation score for all sub–items.

	Z	P	r
Sub 1	-1.342	0.18	0.21
Sub 2	-5.217	<0.001**	0.82
Sub 3	-4.005	<0.001**	0.63
Sub 4	-2.724	0.006	0.43
Sub 5	-2.428	0.015	0.38
Sub 6	-3.464	0.001**	0.55
Sub 7	-2.879	0.004**	0.46
Sub 8	-2.701	0.007	0.43
Sub 9	-5.124	<0.001**	0.81
Sub 10	-3.126	0.002**	0.49

Sub 1, 2, …, 10 represent the scores of the first, second … and tenth sub-items for Fugl-Meyer Assessment. **P<0.005. Bonferroni correction, significance threshold was set at P < 0.005, i.e., 0.05 ÷10 sub-items = 0.005.

Supplementary Table S4. The Shapiro–Wilk test results of all TMS measurements.

	W	df	P
RMT	0.95	40	0.092
MEP	0.87	40	<0.001**
Short-ICI	0.66	40	<0.001**
Long-ICI	0.66	34	<0.001**
Coefficient of variation of MEP	0.92	40	0.006**
Coefficient of variation of short-ICI	0.96	40	0.124
Coefficient of variation of long-ICI	0.91	34	0.006**

RMT, resting motor threshold; MEP, motor evoked potential; ICI, intracortical inhibition. Long-ICI data were collected from 34 patients. **P<0.01.

Supplementary Table S5. Simple linear regression between Z-scored neurophysiological parameters and square-root transformed rehabilitation score changes.

	Total	Proximal	Distal	Whole
RMT	0.10	0.65	1.51	0.80
	(0.332)	(0.427)	(0.226)	(0.780)
MEP	2.39	3.23	0.81	1.31
	(0.130)	(0.080)	(0.374)	(0.260)
Short-ICI	0.56	0.29	1.21	<0.01
	(0.459)	(0.593)	(0.279)	(0.973)
Long-ICI	1.47	1.16	1.67	0.89
	(0.234)	(0.289)	(0.205)	(0.352)
Coefficient of variation of MEP	0.21	0.30	0.02	0.09
	(0.652)	(0.590)	(0.881)	(0.770)
Coefficient of variation of short-ICI	0.27	0.79	0.06	0.71
	(0.610)	(0.381)	(0.813)	(0.406)
Coefficient of variation of long-ICI	0.21	0.16	0.13	0.98
	(0.648)	(0.693)	(0.726)	(0.329)

Data are presented as F-values (P-values). The changes in score were square-root transformed because all rehabilitation effect followed a non-normal distribution. The degrees of freedom for the RMT, MEP, short-ICI, coefficient of variation of MEP, and coefficient of variation of short-ICI was (1, 39), respectively. The degrees of freedom for Long-ICI and coefficient of variation of long-ICI was (1, 33). Long-ICI data were collected from 34 patients.

RMT, resting motor threshold; MEP, motor-evoked potential; ICI, intracortical inhibition.

Supplementary Table S6. Simple logistic regression results between Z-scored neurophysiological parameters and rehabilitation status (improvement/non-improvement).

	Total	Proximal	Distal	Whole
RMT	2.74	0.47	3.39	0.12
	(0.098)	(0.495)	(0.066)	(0.727)
MEP	0.37	1.27	0.22	1.21
	(0.544)	(0.260)	(0.640)	(0.271)
Short-ICI	0.16	3.87	0.95	0.03
	(0.693)	(0.050)	(0.331)	(0.859)
Long-ICI	0.47	0.51	0.30	<0.01
	(0.493)	(0.475)	(0.585)	(0.977)
Coefficient of variation of MEP	0.23	3.34	0.20	0.07
	(0.635)	(0.068)	(0.662)	(0.785)
Coefficient of variation of short-ICI	3.11	2.51	0.49	1.46
	(0.078)	(0.113)	(0.485)	(0.228)
Coefficient of variation of long-ICI	2.84	1.84	2.62	0.70
	(0.092)	(0.176)	(0.105)	(0.402)

Data are presented as χ^2_{LG}-value (P-value). Long-ICI data were collected from 34 patients.

RMT, resting motor threshold; MEP, motor-evoked potential; ICI, intracortical inhibition.

Supplementary Table S7. Simple linear regression between Z-scored neurophysiological parameters and original rehabilitation score changes.

	Total	Proximal	Distal	Whole
RMT	0.27	0.16	0.53	0.06
	(0.607)	(0.689)	(0.472)	(0.809)
MEP	2.21	3.06	0.63	1.37
	(0.145)	(0.089)	(0.431)	(0.249)
Short-ICI	0.56	0.35	1.10	0.13
	(0.459)	(0.560)	(0.300)	(0.721)
Long-ICI	0.01	0.39	0.02	2.78
	(0.921)	(0.536)	(0.899)	(0.105)
Coefficient of variation of MEP	0.001	0.81	0.24	1.12
	(0.975)	(0.374)	(0.627)	(0.297)
Coefficient of variation of short-ICI	0.02	0.57	0.09	0.21
	(0.879)	(0.456)	(0.765)	(0.648)
Coefficient of variation of long-ICI	0.001	0.04	0.03	0.90
	(0.974)	(0.836)	(0.856)	(0.351)

Data are presented as F-values (P-values). The degrees of freedom for the RMT, MEP, short-ICI, coefficient of variation of MEP, and coefficient of variation of short-ICI was (1, 39), respectively. The degrees of freedom for Long-ICI and coefficient of variation of long-ICI was (1, 33). Long-ICI data were collected from 34 patients.

RMT, resting motor threshold; MEP, motor-evoked potential; ICI, intracortical inhibition.

Supplementary Table S8. Simple linear regression between original neurophysiological parameters and original rehabilitation score changes.

	Total	Proximal	Distal	Whole
RMT	0.27	0.16	0.53	0.06
	(0.607)	(0.689)	(0.472)	(0.809)
MEP	2.05	2.92	0.56	1.20
	(0.161)	(0.096)	(0.457)	(0.281)
Short-ICI	0.75	0.47	1.27	0.04
	(0.394)	(0.496)	(0.268)	(0.837)
Long-ICI	0.01	0.38	0.01	2.89
	(0.941)	(0.542)	(0.924)	(0.099)
Coefficient of variation of MEP	0.01	0.93	0.20	0.93
	(0.922)	(0.342)	(0.657)	(0.340)
Coefficient of variation of short-ICI	0.003	0.43	0.12	0.36
	(0.954)	(0.516)	(0.729)	(0.554)
Coefficient of variation of long-ICI	0.002	0.03	0.01	1.07
	(0.968)	(0.856)	(0.927)	(0.309)

Data are presented as F-values (P-values). The degrees of freedom for the RMT, MEP, short-ICI, coefficient of variation of MEP, and coefficient of variation of short-ICI was (1, 39), respectively. The degrees of freedom for Long-ICI and coefficient of variation of long-ICI was (1, 33). Long-ICI data were collected from 34 patients.

RMT, resting motor threshold; MEP, motor-evoked potential; ICI, intracortical inhibition.

Supplementary Table S9. Simple logistic regression results between original neurophysiological parameters and rehabilitation status (improvement/non-improvement).

	Total	Proximal	Distal	Whole
RMT	2.74	0.47	3.39	0.12
	(0.098)	(0.495)	(0.066)	(0.727)
MEP	0.37	1.27	0.022	1.21
	(0.544)	(0.260)	(0.640)	(0.271)
Short-ICI	0.16	3.87	0.95	0.03
	(0.693)	(0.050)	(0.331)	(0.859)
Long-ICI	0.47	0.51	0.30	0.001
	(0.493)	(0.475)	(0.585)	(0.977)
Coefficient of variation of MEP	0.23	3.34	0.19	0.07
	(0.635)	(0.068)	(0.662)	(0.785)
Coefficient of variation of short-ICI	3.11	2.51	0.49	1.46
	(0.078)	(0.113)	(0.485)	(0.228)
Coefficient of variation of long-ICI	2.84	1.84	2.62	0.70
	(0.092)	(0.176)	(0.105)	(0.402)

Data are presented as χ^2_{LG}-value (P-value). Long-ICI data were collected from 34 patients.

RMT, resting motor threshold; MEP, motor-evoked potential; ICI, intracortical inhibition.

Supplementary Table S10. Generalized linear model results between Z-scored neurophysiological parameters and original rehabilitation score changes.

	χ^2_{LG}	df	P
Total	4.23	7	0.754
Proximal	5.45	7	0.605
Distal	3.78	7	0.804
Whole	5.92	7	0.549

Data were collected from 34 patients.

Supplementary Table S11. Generalized linear model results between original neurophysiological parameters and original rehabilitation score changes.

	χ^2_{LG}	df	P
Total	4.12	7	0.766
Proximal	5.26	7	0.628
Distal	3.74	7	0.809
Whole	6.14	7	0.523

Data were collected from 34 patients.

Supplementary Table S12. Binary logistic regression results between original neurophysiological parameters and rehabilitation status (improvement/non-improvement).

	χ^2_{LG}	P	AUC	R^2	AIC	BIC
Total	14.86	0.038*	0.84	0.47	47.80	60.01
Proximal	16.71	0.019*	0.87	0.52	45.36	57.57
Distal	10.99	0.139	0.83	0.38	49.16	61.37
Whole	5.17	0.639	0.71	0.19	57.84	70.05

Data were collected from 34 patients. *P<0.05.

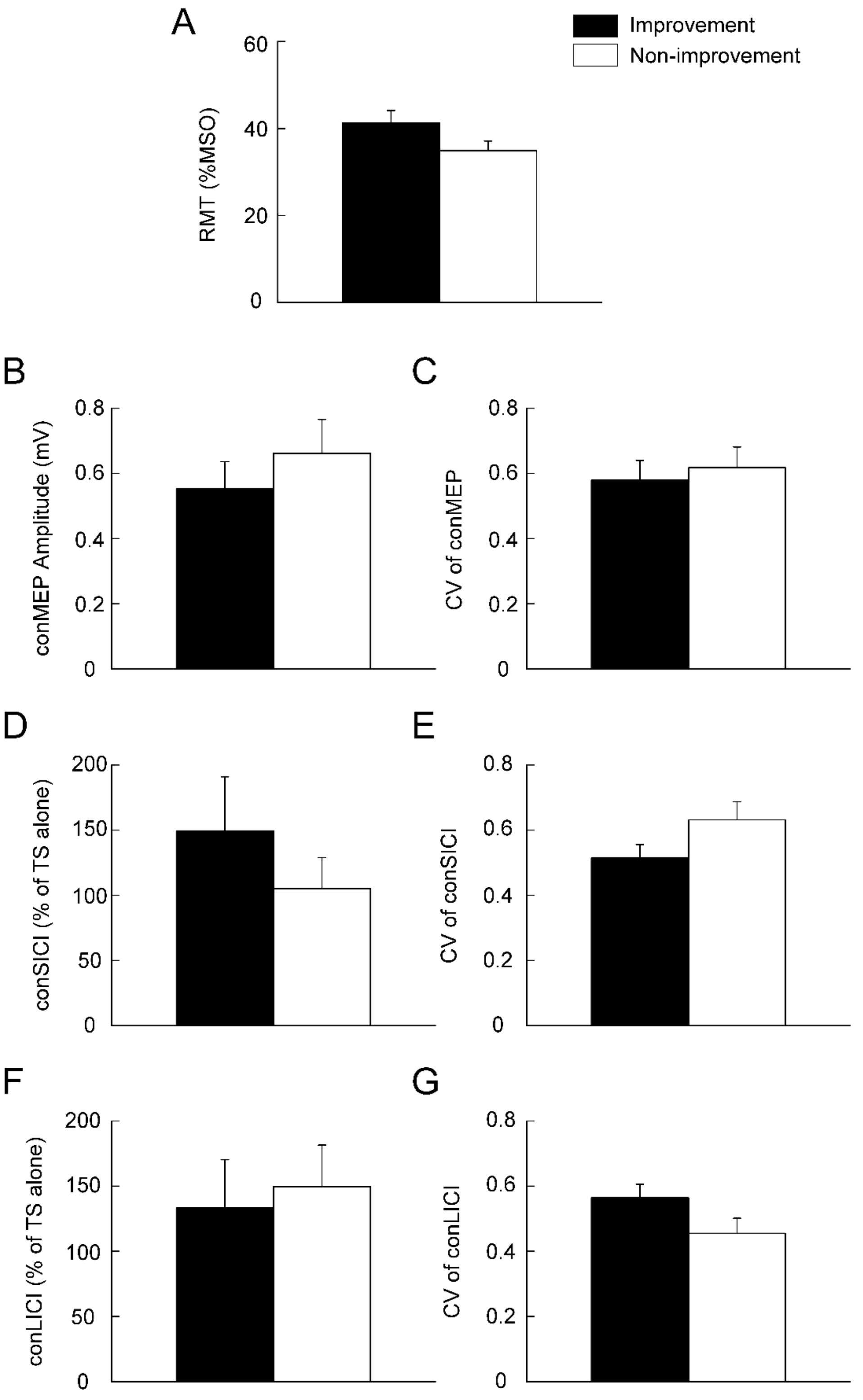

Figure S1. Between-group comparisons of neurophysiological parameters. (A) to (G) depict the following measures in order: RMT, MEP amplitude, coefficient of variation of MEP, short-ICI, coefficient of variation of short-ICI, long-ICI, and coefficient of variation of

long-ICI. Data in (A) to (E) are from 40 patients, while data in (F) and (G) are from 34 patients. Data are presented as mean ± standard error. RMT, resting motor threshold; MSO, maximum stimulus output; MEP, motor-evoked potential; CV, coefficient of variation; ICI, intracortical inhibition.

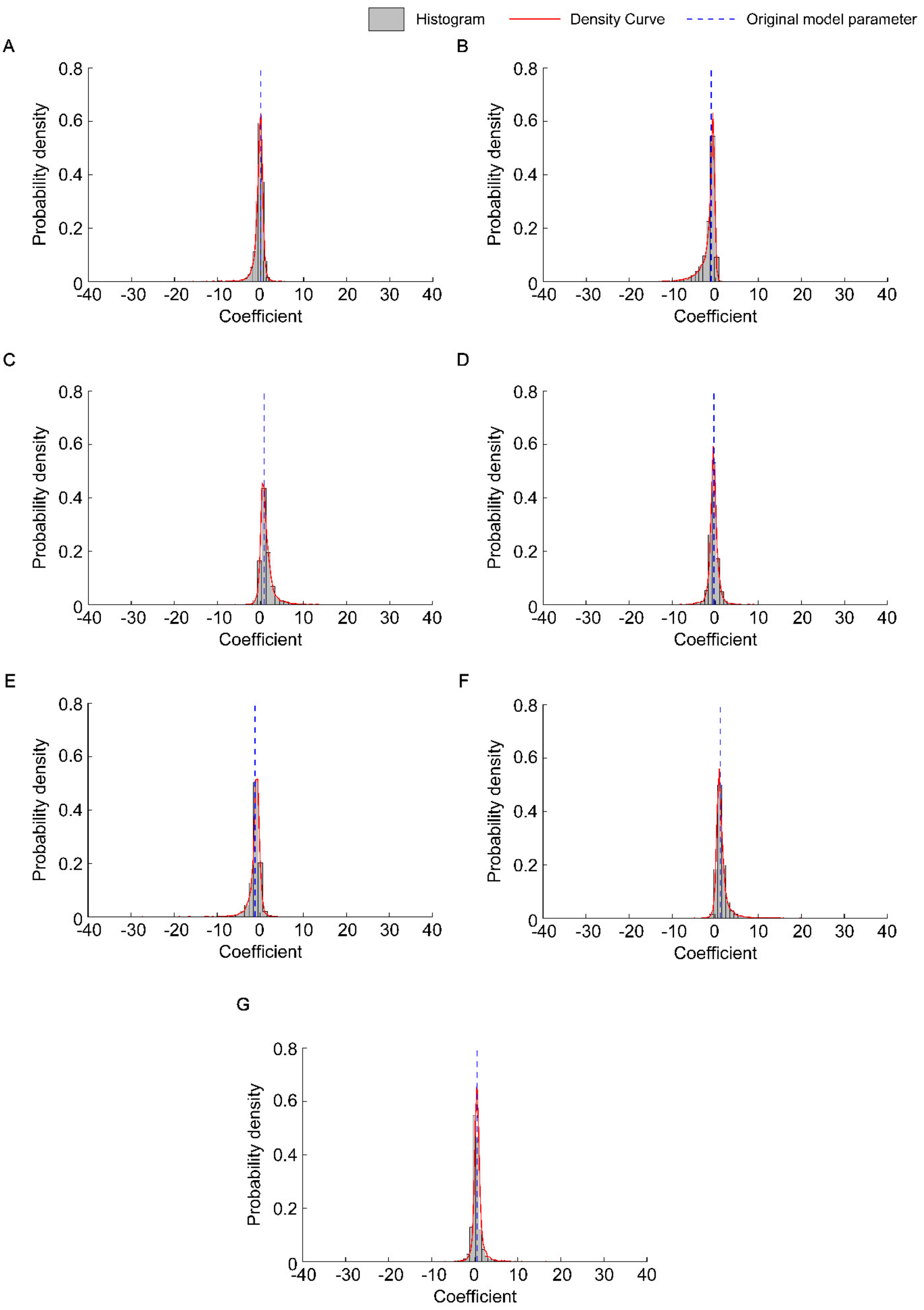

Figure S2. Distribution plots of coefficient from bootstrap resampling (n = 5000) for the

Proximal Recovery Prediction Model Panels A-G correspond to the following predictors: RMT, MEP, long-ICI, coefficient of variation of MEP, coefficient of variation of short-ICI, coefficient of variation of long-IC and the constant term, respectively. The significant predictor short-ICI from the original model has been reported in the main text (Figure 5). The vertical axis shows probability density, calculated as frequency divided by (total sample count × bin width). The horizontal axis represents the coefficient values. The red curve indicates the fitted probability density curve, while blue dashed lines mark the coefficient position of each predictor from the original model. RMT, resting motor threshold; MEP, motor-evoked potential; ICI, intracortical inhibition.

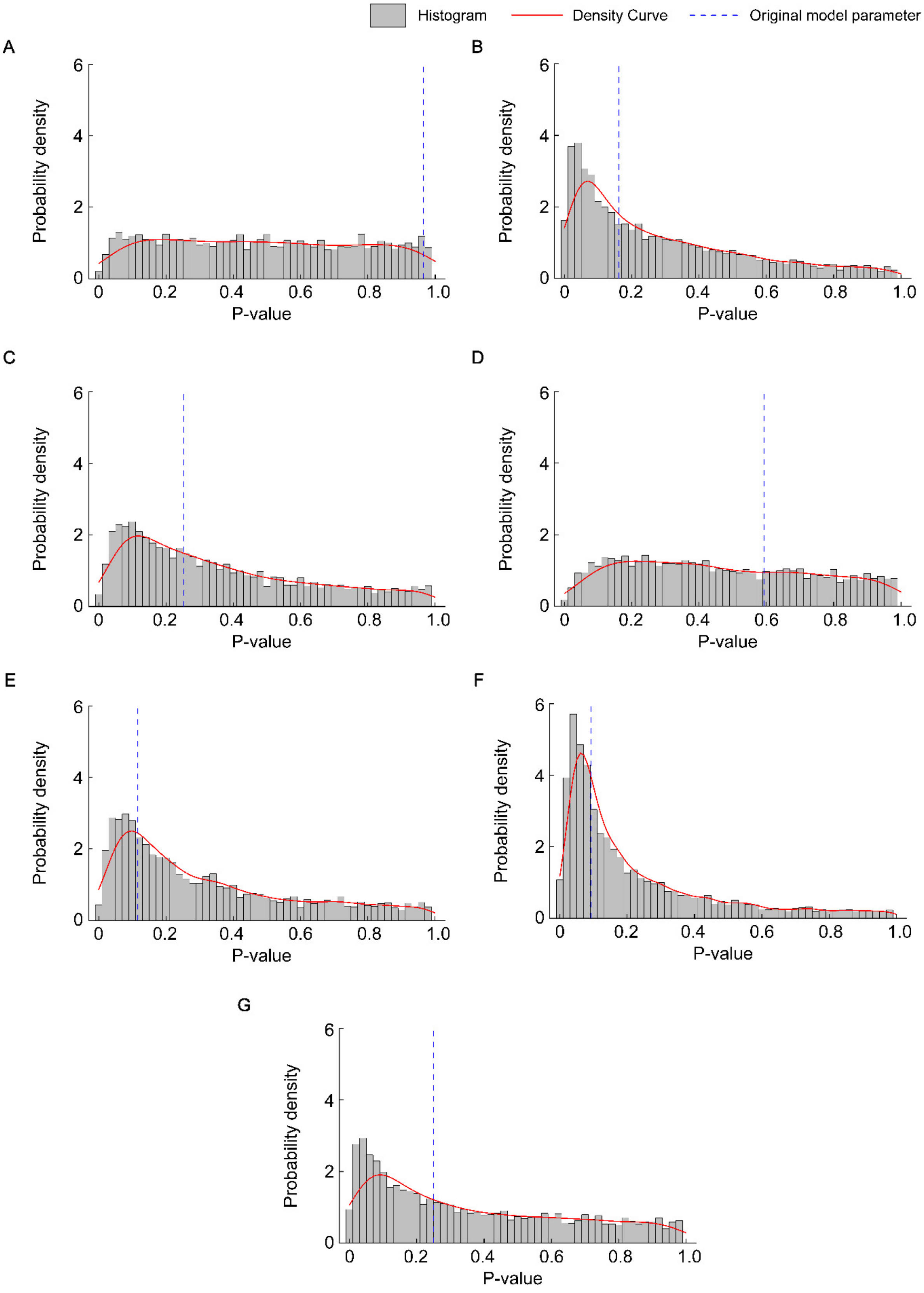

Figure S3. Distribution plots of P-value from bootstrap resampling (n = 5000) for the

Proximal Recovery Prediction Model. Panels A-G correspond to the following predictors: RMT, MEP, long-ICI, coefficient of variation of MEP, coefficient of variation of short-ICI, coefficient of variation of long-IC and the constant term, respectively. The significant predictor short-ICI from the original model has been reported in the main text (Figure 5). The vertical axis shows probability density, calculated as frequency divided by (total sample count × bin width). The horizontal axis represents the P-values. The red curve indicates the fitted probability density curve, while blue dashed lines mark the P-value position of each predictor from the original model. RMT, resting motor threshold; MEP, motor-evoked potential; ICI, intracortical inhibition.
